# Single-bacterium RNA-seq protocol to uncover heterogeneous expression of coding and noncoding genes in *Bacteroides thetaiotaomicron*

**DOI:** 10.1016/j.xpro.2026.104651

**Published:** 2026-07-01

**Authors:** Elise Bornet, Alexander J. Westermann

**Affiliations:** 1 Department of Microbiology, Biocenter, University of Würzburg, Würzburg, Germany; 2 Helmholtz Institute for RNA-based Infection Research, Helmholtz Centre for Infection Research, Würzburg, Germany; 3 Cluster for Nucleic Acid Sciences and Technologies – NUCLEATE, Josef-Schneider-Str. 2 / D15, 97080 Würzburg, Germany

**Keywords:** Single cell, RNAseq, Microbiology, Gene expression

## Abstract

Single-cell RNA sequencing is increasingly applied to bacterial model species, but a dedicated technique for anaerobic microbiota members of the *Bacteroidota* phylum has been lacking. Here, working with *Bacteroides thetaiotaomicron*, we describe experimental steps for transcriptome stabilization, fluorescence-activated cell sorting (FACS) collection, and optimized lysis of single cells from this group of organisms. We detail procedures for reverse transcription of RNAs via the multiple annealing and dC-tailing-based quantitative single-cell RNA sequencing (MATQ-seq) protocol, sensitive Cas9-based depletion of ribosomal sequences, and cDNA library generation.

For complete details on the use and execution of this protocol, please refer to Bornet et al.^1^

## Highlights

• Instructions for FACS isolation of single *Bacteroides thetaiotaomicron* cells
• Steps for MATQ-seq-based reverse transcription of the transcriptomes of single *Bacteroides* cells
• Guidance on Cas9-based rRNA depletion from *Bacteroides* single-cell cDNA libraries

## Abstract (editor highlights)

Publisher’s note: Undertaking any experimental protocol requires adherence to local institutional guidelines for laboratory safety and ethics.

## Graphical abstract

**Figure:**
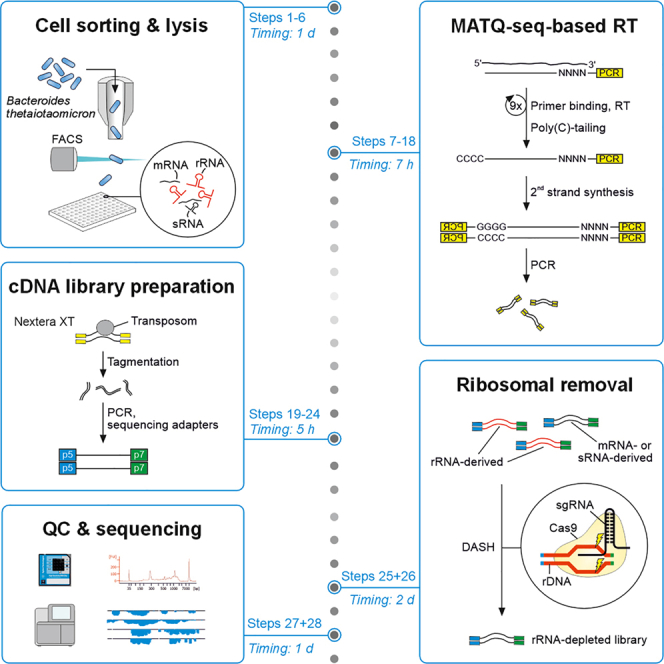

## Before you begin

Transcriptomic approaches increasingly take bacterial variability into account and aim at the analysis of defined sub-populations of cells—be it through the use of reporter strains that inform about a specific trait such as growth rate^2^ or by collecting bacteria populating specific host niches.^3^ However, transcriptomic heterogeneity may pertain even between similarly replicating or co-localizing bacteria.^4^ To uncover bacterial cell-to-cell variability and understand its role for population fitness, single-cell technologies are imperative. Single-cell RNA-seq analysis, widely used for the study of eukaryotic cells,^5^ is now increasingly applied to prokaryotes.^6^^,^^7^^,^^8^ Although not yet on a genome-wide scale, the few hundred bacterial genes detected per single cell by recent protocols^9^^,^^10^^,^^11^^,^^12^^,^^13^^,^^14^^,^^15^^,^^16^^,^^17^ allow for unbiased, explorative analyses and represent a huge leap forward as compared to profiling a small number of preselected bacterial genes or traits. In this regard, the MATQ-seq^10^^,^^18^ approach has gained attraction, as it provides high coverage of the transcriptome of single bacteria and detects transcripts relatively independent of their length and coding potential. Besides, unlike split-pool-based alternatives,^9^^,^^11^ MATQ-seq is compatible with FACS-based collection of single bacterial cells, allowing an enrichment of specific cell types prior to sequencing. MATQ-seq-based single-bacterium RNA-seq was originally established in Gammaproteobacteria; specifically in *Escherichia coli*, *Salmonella enterica*, and *Pseudomonas aeruginosa*.^10^^,^^19^ The physico-chemical differences between the cell membranes and varying RNA contents of these species prevented the establishment of a universal, one-size-fits-all protocol, implying that MATQ-seq would have to be empirically optimized for any future target organism. In particular, the method had not previously been applied to obligate anaerobic bacteria, such as members of the *Bacteroidota*, i.e., the bacterial phylum predominating in the human gut microbiota. Given these organisms’ intrinsic vulnerability to ambient oxygen and several characteristic cellular features—including a dynamic surface structure^20^^,^^21^^,^^22^ and morphological heterogeneity^1^^,^^23^^,^^24^—the adoption of MATQ-seq in *Bacteroidota* was expected to require special optimizations.

**Figure 1:**
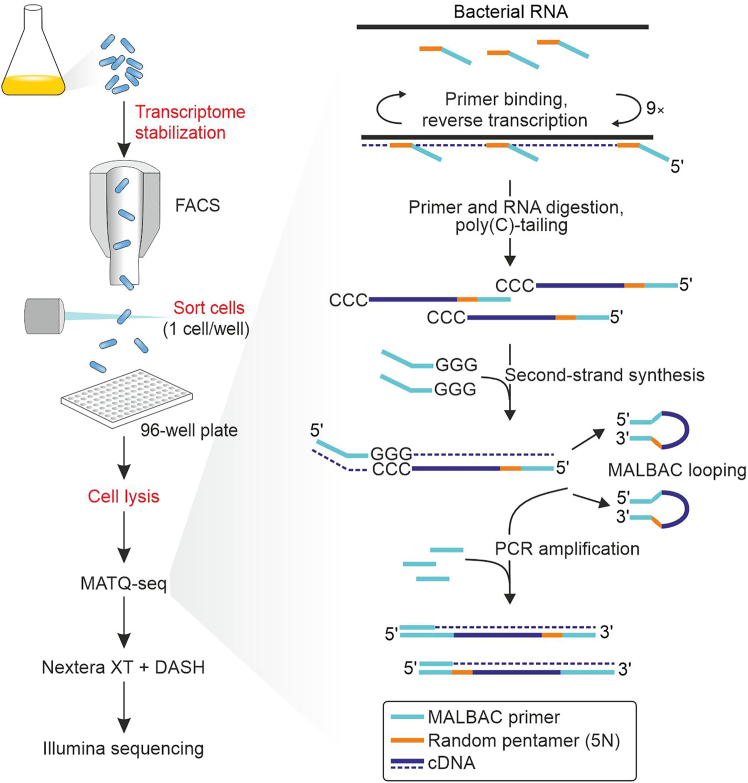
Schematic of the *Bacteroides* single-cell RNA-seq protocol The steps where our protocol diverges from similar protocols tailored to *Enterobacteriaceae*^25^^,^^26^ are highlighted in red and may require further optimization when transferring the protocol to yet other bacterial species. Specifically, testing different concentrations of RNA*later* may be necessary to efficiently stabilize the transcriptome of cells without interfering with downstream steps. It is also important to tailor the FACS settings to the target organism. Finally, a crucial step involves bacterial cell lysis and for *B*. *thetaiotaomicron*, mechanical lysis by sonication yielded the best results. MALBAC, Multiple Annealing and Looping-Based Amplification Cycles; DASH, Depletion of Abundant Sequences by Hybridization.

The protocol below describes the specific experimental steps of our optimized MATQ-seq protocol to sequence the transcriptome of single *B*. *thetaiotaomicron* cells (Figure 1). The full procedure from sampling to sequencing can be conducted in ∼5 days. The step-by-step protocol provided below refers to the manual pipetting of the respective reaction mixes. However, we indicated where automated pipetting using a liquid-handling robot may be applied, if such a system is available. There are certain steps (namely the preparation of the 96-well plate for bacterial cell-sorting and the reverse transcription according to the MATQ-seq protocol) that need to be performed in a sterile room, and all surfaces and equipment should be thoroughly cleaned with ethanol and RNase Zap before you begin.

### Innovation

Our protocol involves transcriptome stabilization to minimize oxygen stress-induced gene expression changes and the optimization of sorting and lysis conditions to *B*. *thetaiotaomicron* cells. For example, unlike previous bacterial MATQ-seq protocols, we omit lysozyme treatment, using sonication alone for lysis. The workflow also implements Cas9-mediated cleavage of cDNA fragments derived from ribosomal RNA (rRNA),^27^ reducing the corresponding reads from ∼80% to ∼20%, while increasing informative reads from protein-coding and regulatory RNAs. We have previously used this protocol to profile the transcriptomes of small populations (comprised of 100 individual bacteria) and single cells of *B*. *thetaiotaomicron*^1^ as well as on a mixed consortium of *B*. *thetaiotaomicron* and *E*. *coli* within a microfluidic device,^28^ suggesting that it generally lends itself for the measurement of gene expression in low-biomass *Bacteroides* samples.

### Preparation of *Bacteroides thetaiotaomicron* cultures

**Timing:** Variable (∼4 days)

***Note:*** Generally, *B*. *thetaiotaomicron* strains are cultured on supplemented Brain-Heart-Infusion supplemented (BHIS) agar plates or in liquid Tryptone Yeast Glucose (TYG) medium, in an anaerobic atmosphere (80% N_2_, 10% CO_2_, 10% H_2_) at 37°C.

1. Plating *B. thetaiotaomicron.*
  a. From a cryo (glycerol) stock, streak *B. thetaiotaomicron* on a BHIS plate.
  b. Incubate in an anaerobic chamber at 37°C for 2-3 days or until single colonies emerge.

***Note:*** Plates can be kept for up to 7 days. Thereafter, we recommend re-streaking from the glycerol stock.

2. Liquid culture growth.
  a. Inoculate a single colony of *B. thetaiotaomicron* in 5 mL of degassed TYG.
    ***Note:*** Degas TYG medium by placing it in the anaerobic chamber for a minimum of 4 h.
  b. Incubate the culture for ∼16 h at 37°C in an anaerobic atmosphere without shaking.
  c. The next morning, subculture the overnight culture 1:100 in degassed TYG.
  d. Grow until the culture reaches its intended growth phase.
    ***Note:*** In TYG, an OD_600_ of 0.3 (reached after ∼4 h of culturing in the described way) corresponds to early exponential phase, an OD_600_ of 2.0 (∼8 h) to mid-exponential phase, and an OD_600_ >4.0 (∼10 h) to late exponential phase (Figure 2). As a reference point, when applying this protocol to generate single-bacterium RNA-seq libraries from *B*. *thetaiotaomicron* cells harvested at mid-exponential phase, the overall success rate was 96%.
    **Figure 2:**
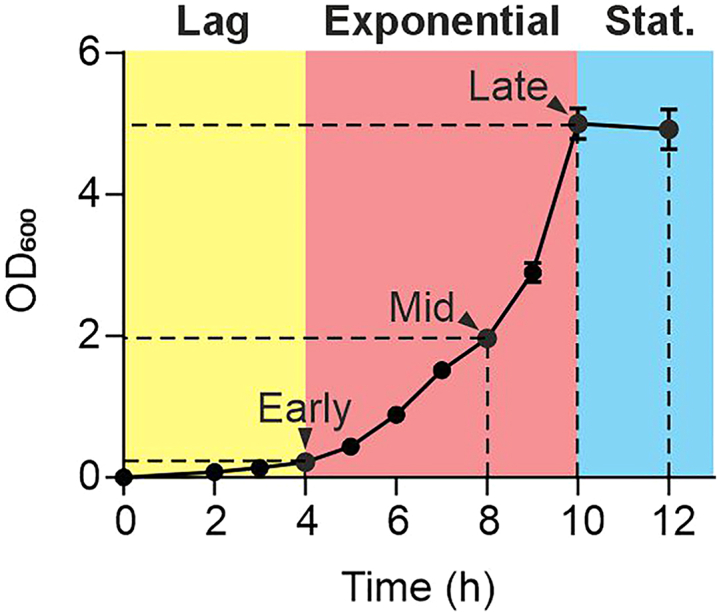
Growth curve of a *B*. *thetaiotaomicron* culture in TYG (average of 5 biological replicates) Figure reproduced from.^1^

### Preparation of positive control RNA samples

**Timing:** Variable (∼2–3 days)

3. RNA extraction and DNase treatment.
  a. Extract total RNA from *B. thetaiotaomicron* following the hot-phenol RNA extraction protocol^29^ and elute in nuclease-free water.
  b. Measure the total RNA concentration on a NanoDrop instrument.
  c. Prepare the DNase mix solution in a total volume of 50 μL, as follows:

**Table:** 

Reagent	Amount
DNase I buffer (10x)	5 μL
Superase-In RNase Inhibitor (20 U/μL)	0.5 μL
DNase I (Thermo Fisher Scientific) (1 U/μL)	1 U/μg of RNA
RNA in nuclease-free water	5-30 μg
Nuclease-free water	Adjust to 50 μL
  d. Incubate for 45 min at 37°C.
  e. Add 50 μL of nuclease-free water.
  f. Proceed to RNA extraction with phenol/chloroform/isoamyl alcohol (PCI) and phase-lock gel tubes.
    **CRITICAL:** When handling PCI, wear gloves and work under a chemical hood.
    i. Add 100 μL of PCI to the tube in step 3e.
    ii. Transfer to a phase-lock gel tube.
    iii. Centrifuge for 12 min, at 15°C and 15,600 × g.
    iv. Transfer the aqueous phase (upper layer) into a new tube.
    v. Add 2 volumes of 30:1 EtOH:NaOAc, pH 6.5.
    vi. Precipitate for 2-16 h at −20°C.
    vii. Centrifuge for 30 min, at 4°C and 15,600 × g.
    viii. Discard the supernatant and wash the RNA pellet with 200 μL of ice-cold 75% ethanol.
    ix. Centrifuge for 10 min, at 4°C and 15,600 × g.
    x. Discard the supernatant and air-dry the pellet.
    xi. Resuspend the pellet in 30 μL of nuclease-free water.
    xii. Dissolve the pellet by incubating for 5 min at 65°C with gentle shaking at 700-900 rpm.
    xiii. Measure the RNA concentration using a NanoDrop device.
4. Prepare the positive control aliquots for the MATQ-seq protocol.
  a. Dilute the DNase-treated RNA sample to a concentration of 100 pg/μL and split into 20 μL aliquots.

***Note:*** These aliquots should be stored at −80°C until they are used.

### Preparation of reagents and stocks

**Timing:** 20 min

5. Resuspend the oligonucleotides (required in step 13) in nuclease-free water to a concentration of 100 μM and split the different oligonucleotides into each 20 μL aliquots, in 1.5 mL LoBind Eppendorf tubes.
6. Prepare 5 mL of fresh 50% RNA*later* solution by mixing pure RNA*later* with PBS at a 1:1 ratio [v/v].

***Note:*** RNA*later* is relatively viscous and should be pipetted with caution.

### Institutional permissions (if applicable)

Work with live *B*. *thetaiotaomicron* is conducted in a biosafety level 2 laboratory.

## Key resources table

**Table:** 

REAGENT or RESOURCE	SOURCE	IDENTIFIER
**Bacterial and virus strains**		

*Bacteroides thetaiotaomicron* VPI-5842 (WT)	DSM	2079
*Bacteroides thetaiotaomicron* GFP+	Whitaker et al.^30^	N/A

**Chemicals, peptides, and recombinant proteins**		

Brain-Heart-Infusion (BHI) agar	Carl Roth	X915.1
Cas9 nuclease	New England Biolabs	MO386T
dCTPs	Invitrogen	10217016
Deepvent DNA polymerase/Thermopol buffer	New England Biolabs	M0259L
DNase I	Thermo Fischer Scientific	EN0521
dNTPs	New England Biolabs	N0447L
Ethanol Absolute for analysis	Merck	1.00983.2511
Glucose	Merck	1.08337.1000
Hemin	Carl Roth	7629.2
L-cysteine	Carl Roth	3467.3
Nuclease-free water	Ambion	AM9916
Proteinase K	New England Biolabs	P8107S
10x PBS	Gibco	70011044
Phenol/chloroform/isoamyl alcohol (PCI)	Carl Roth	A156.1
Recombinant RNase inhibitor	Takara	2313A
RNA*later*	Qiagen	76104
RNase H	New England Biolabs	M0297L
RNase If	New England Biolabs	M0243S
SuperScript IV reverse transcriptase/buffer/DTT	Thermo Fischer Scientific	18090010
Superase-In RNase inhibitor	Thermo Fischer Scientific	AM2694
T4 DNA polymerase	New England Biolabs	M0203L
Terminal transferase & buffer	New England Biolabs	M0315S
Tryptone	Carl Roth	8952.3
10x lysis buffer	Takara	635013
Yeast extract	Carl Roth	2363.3
MgSO_4_.7H_2_O	Carl Roth	8283.2
KH_2_PO_4_	Carl Roth	P018.5
K_2_HPO_4_	Carl Roth	6875.3
NaCl	Carl Roth	3957.1
NaOAc	Carl Roth	6779.2
CaCl_2_	Carl Roth	CN93.1
NaHCO_3_	Carl Roth	6885.1
NaOH	Alfa Aesar	A12173

**Critical commercial assays**		

Agilent high-sensitivity DNA kit	Agilent	5067–4626
Ampure XP beads	Beckman Coulter	A63881
KAPA HiFi HotStart ReadyMix	Roche sequencing store	KK2602/SKU:7958935001
MEGAshortscript kit	ThermoFischer Scientific	AM1354
Monarch RNA cleanup kit	NEB	T2050S
Nextera XT kit	Illumina	FC-131-1096
Qubit dsDNA HS assay kit	Invitrogen	Q32854
TE buffer	Invitrogen	AM9849
Oligo Clean & Concentrator	Zymo Research	D4060
RNAseZap	Invitrogen	10708345
FACSFlow	BD Biosciences	342003
Unique Dual Indexes (UDI)	Illumina	20091654

**Deposited data**		

GEO	N/A	GSE322907

**Oligonucleotides**		

GTG AGT GAT GGT TGA GGA TGT GTG GAG NNN NN TTTTTTTTTTTTTTTTTTTT	IDT (HPLC purified)	GAT27 poly(dT)
GTG AGT GAT GGT TGA GGA TGT GTG GAG NNN NNG GG	IDT (HPLC purified)	GAT27 5N3G
GTG AGT GAT GGT TGA GGA TGT GTG GAG NNN NNT TT	IDT (HPLC purified)	GAT27 5N3T
GAT GGT TGA GGA TGT GTG GAG NNN NNN GGG	IDT (HPLC purified)	GAT21 6N3G - MATQ-seq
GTG AGT GAT GGT TGA GGA TGT GTG GAG	IDT (HPLC purified)	GAT27 PCR - MATQ-seq
GGCATACTCTGCGACATCGT	IDT (standard, desalting)	Reverse primer for fill-in of *B*. *thetaiotaomicron* DASH 651 oligo pool
AATGATACGGCGACCACCGA	IDT (standard, desalting)	p5 adaptor Illumina DASH amplification primer (forward)
CAAGCAGAAGACGGCATACGAGAT	IDT (standard, desalting)	p7 adaptor Illumina DASH amplification primer (reverse)
DASH 651 oligo pool	Prezza et al.^27^	For list of oligonucleotides to generate single-guide RNAs, see Table S1

**Software and algorithms**		

BBDUK	Bushnell et al.^31^	sourceforge.net/projects/bbmap/
Bowtie2	Langmead et al.^32^	sourceforge.net/bowtie2/manual.shtml
Fastqc	Andrews et al.^33^	https://github.com/s-andrews/FastQC
featureCounts	Liao et al.^34^	sourceforge.net/featureCounts.html

**Other**		

Anaerobic chamber	Coy Laboratory Product	N/A
96-well PCR plates (non-skirted)	Brand	781368
Accudrop beads	BD Biosciences	345249
Fluorescence-activated cell sorter	BD Biosciences	FACS Aria III
I.DOT	Dispendix	N/A
I.DOT Pure Plate 100	Dispendix	D16110021801
I.DOT Pure Plate 200	Dispendix	D16110021807
LoBind 1.5 mL Eppendorf tubes	Eppendorf	41121702
LoBind 2 mL Eppendorf tubes	Eppendorf	41103037
5 mL Eppendorf tubes	Eppendorf	0030119401
Magnetic racks	ThermoFisher Scientific	DynaMag-96
Microseal ‘B’ seal (adhesive film)	BioRad	MSB1001B
Microseal ‘F’ seal (foil)	BioRad	MSF1001B
Phase-lock gel tubes (5PRIME Phase Lock Gel Heavy, 2 mL)	VWR	2302830
Sonicator	Bendelin	Sonorex Digitec DT 52
Combispin	BioSan	FVL-2400N
Centrifuge	Eppendorf	5415R
Vortexer	Scientific Industries	Vortex Genie 2
Head set for vortex	Merck	Z258431
Mini plate spinner centrifuge	Labnet	MPS 1000
Thermocycler	BioRad	T100 Thermal Cycler
Bioanalyzer	Agilent	2100 Bioanalyzer
Qubit 4 fluorometer	Thermo Fischer Scientific	Q33238
Qubit Flex Fluorometer	Thermo Fischer Scientific	Q33327
NanoDrop	Thermo Fisher Scientific	ND-2000C

## Materials and equipment

**Table:** Tryptone Yeast Glucose (TYG) medium

Reagent	Final concentration	Amount
Tryptone	20 g/L	20 g
Yeast extract	10 g/L	10 g
Glucose (anhydrous or monohydrate)	5 g/L or 5.5 g/L	5 g or 5.5 g
L-cysteine	8.25 mM	1 g
ddH_2_O	N/A	890 mL
Hemin	1%	10 mL
Salt solution	4%	40 mL
CaCl_2_	0.072 mM	40 mL
NaHCO_3_ (10%)	0.2%	20 mL
**Total Volume**	**N/A**	**1 L**

Filter-sterilize with a 0.2 μm sterile vacuum filter. Degas in an anerobic environment for ≥4 h prior usage. TYG medium can be stored in an anaerobic chamber and kept for up to 7 days.

**Table:** Salt solution

Reagent	Final concentration	Amount
MgSO_4_.7H_2_O	1.95 mM	0.24 g
KH_2_PO_4_	7. 43 mM	0.5 g
K_2_HPO_4_	5.74 mM	0.5 g
NaCl	34.2 mM	1 g
ddH_2_O	N/A	500 mL
**Total volume**	**N/A**	**500 mL**

Filter-sterilize with a 0.2 μm sterile filter and dispense in 50 mL Falcon tubes. Store at 4°C.

**Table:** Calcium chloride (CaCl_2_)

Reagent	Final concentration	Amount
CaCl_2_	1.8 mM	0.1 g
ddH_2_O	N/A	500 mL
**Total volume**	**N/A**	**500 mL**

Filter-sterilize with a 0.2 μm sterile filter and dispense in 50 mL Falcon tubes. Store at 4°C.

**Table:** Sodium bicarbonate (NaHCO_3_), 10%

Reagent	Final concentration	Amount
NaHCO_3_	100 g/L	5 g
ddH_2_O	N/A	50 mL
**Total volume**	**N/A**	**50 mL**

Warm shortly to 50°C to dissolve. Filter-sterilize with a 0.2 μm sterile filter and store at 4°C.

**Table:** Hemin solution

Reagent	Final concentration	Amount
Hemin	0.5 g/L	50 mg
NaOH (1 M)	10 mM	1 mL
ddH_2_O	N/A	99 mL
**Total volume**	**N/A**	**100 mL**

Filter-sterilize with a 0.2 μm sterile filter. The solution is light-sensitive. Store in a brown glass bottle at 4°C for up to 2 weeks.

**Table:** Brain-Heart-Infusion supplemented (BHIS) medium

Reagent	Final concentration	Amount
Brain-Heart-Infusion Agar	37 g/L	37 g
L-cysteine	8.25 mM	1 g
Hemin	1%	10 mL
ddH_2_O	N/A	970 mL
NaHCO_3_ (10%)	2 g/L	20 mL
**Total volume**	**N/A**	**1 L**

Autoclave the medium and pour plates.

## Step-by-step method details

### Establish FACS settings and gating strategy

**Timing: Variable (∼1 day)**

This section comprises of two parts: the establishment of the FACS settings, including the gating strategy (steps 1–5) and the actual sort-collection of bacterial cells for downstream RNA sequencing (step 6). We recommend a pilot experiment using fluorescent *Bacteroides* cells to help define the optimal FACS gates.

1. Set up the FACS instrument.
  ***Note:*** To ensure a stable stream and accurate sorting, the below steps should be completed about 2 h before the culture reaches the desired OD_600_.
  a. Turn on the FACS instrument and set the cooling block to 4°C.
  b. Insert the 70 μm nozzle.
  c. Perform an automated drop delay using FACS Accudrop Beads, according to the manufacturer’s recommendation.
  d. Let the stream stabilize for at least 30 min at a flow rate of 4.
2. Prepare a 96-well plate for bacterial sorting.
  ***Note:*** The plate should be freshly prepared under a sterile hood or a clean bench about 1 h before the culture reaches the desired OD_600_, to allow for sufficient time for setting up the FACS.
  a. Mix the following components and adjust the volume to the number of wells.
    ***Note:*** It is recommended to account for 5 spare reactions.

**Table:** 

Reagent	Amount
10x lysis buffer (Takara)	0.26 μL
Recombinant RNase inhibitor (Takara)	0.03 μL
PBS (10x)	0.26 μL
Nuclease-free water	2.05 μL
  b. Dispense each 2.6 μL of the above mix in individual wells of a 96-well plate.
  c. Spin the plate down for 5 s, on a mini plate spinner centrifuge.
  d. Seal the plate with Microseal ‘B’.
    ***Note:*** This plate seal tolerates low temperatures and remains adhesive down to −40°C.
    **CRITICAL:** Keep the plate on ice until further processing.
3. Collect GFP-expressing *B. thetaiotaomicron* cells (GFP+ strain;^30^) from a liquid culture in a defined growth phase.
  a. At the desired OD_600_, collect 1 OD-equivalent of cells in a tube (i.e., if collecting cells at OD_600_ = 1.0, collect 1 mL of the bacterial culture; at OD_600_ = 2.0, collect 500 µL).
  b. Centrifuge for 4 min at 1,300 × *g* and at ∼21°C inside the anaerobic chamber.
    **CRITICAL:** To minimize transcriptional changes once the cells have been harvested—e.g. the induction of an oxygen stress response—it is important to work quickly, on ice, and under anaerobic conditions, until the RNA samples will be stabilized in RNA*later*.
  c. Discard the supernatant and wash the pellet once with 1 mL of ice-cold 1x PBS.
    ***Note:*** When establishing the FACS settings in a pilot experiment with the help of a fluorescent strain, it may be required to incubate the cell sample for some time in presence of atmospheric oxygen, for the fluorescent protein to fold into its active form (e.g. in case of the *B*. *thetaiotaomicron* GFP+ strain, we incubated for ≥15 min). However, once the sorting conditions are established, switch to the corresponding wild-type strain and omit this incubation step, keeping the oxygen exposure to a minimum.
  d. Resuspend the cells in 1 mL of 50% RNA*later* (1:1 [v/v] RNA*later*:1x PBS).
    ***Note:*** The concentration of RNA*later* can be adjusted when necessary. We found that 100% RNA*later* rendered cDNA library generation for *B*. *thetaiotaomicron* inefficient.^1^ In contrast, 100% RNA*later* worked well to stabilize *S.enterica* cells prior to MATQ-seq-based single-bacterium RNA-seq analysis.^19^ One difference may lie in the cell envelope structure and RNA accessibility between *Salmonella* (Gram-negative bacterium) and *Bacteroides* (also Gram-negative, but with a distinct cell envelope composition^20^^,^^21^^,^^22^). High RNA*later* concentrations might alter *Bacteroides* membrane properties, affecting permeabilization or causing RNA loss. Diluting to 50% likely strikes a balance for *Bacteroides* between RNA stabilization and compatibility with single-cell RNA-seq steps.
    **CRITICAL:** Keep the cells on ice until further processing!
4. Establish the FACS settings to sort *Bacteroides* cells.
  a. Prepare a negative control consisting of the sample buffer (i.e., 50% RNA*later*) diluted in 49 volumes of 1× ice-cold PBS to a final volume of 1 mL in a 2 mL LoBind Eppendorf tube.
    ***Note:*** This negative control is included to gate against background noise (e.g. salt particles) (Figure 3A).
    **Figure 3:**
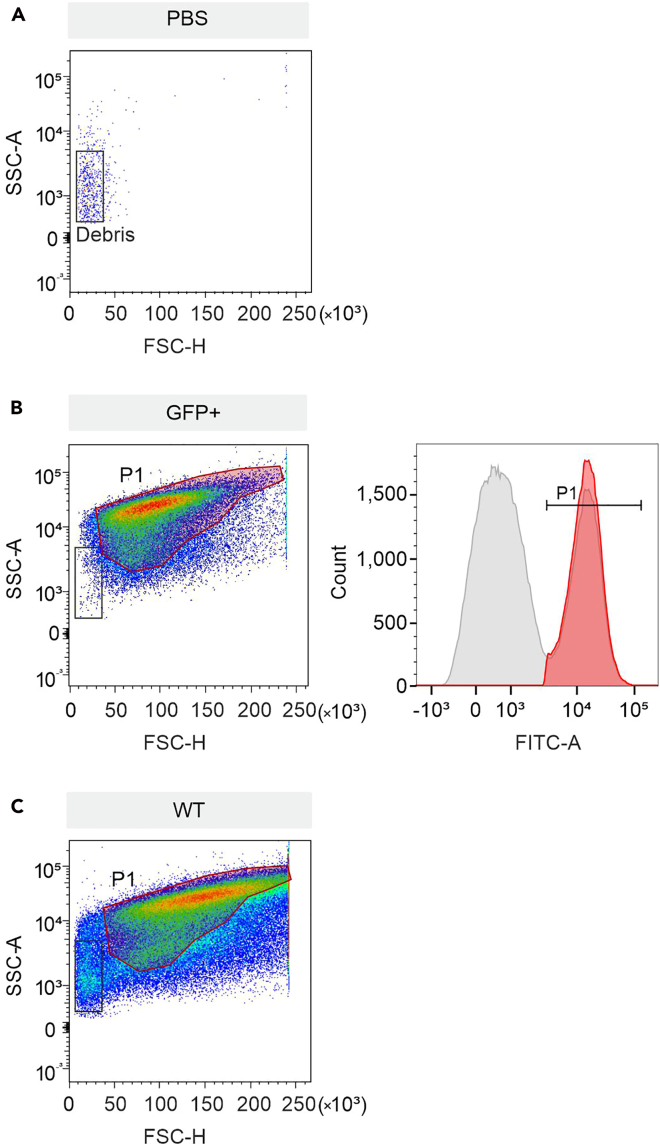
Setting the gates for *Bacteroides* cell-sorting (A) Representative FACS scatter plot of a blank sample (PBS only) in the forward/side scatter (FSC/SSC) channels. (B) Representative FSC/SSC plot of a constitutively GFP-expressing (GFP+) *B. thetaiotaomicron* strain (left) and histogram of the fluorescence intensity of individual events in the FITC channel (right). Intact bacterial cells are selected for sorting based on their high FITC-A signal (gate ‘P1’) and counter-selected against debris and salt particles based on the ‘Debris’ gate around the negative control (panel a). (C) Using the gating strategy defined in panel b, *B*. *thetaiotaomicron* wild-type (WT) cells are collected. Shown here is a representative FACS scatter plot in the FSC/SSC channels.
  b. Dilute the bacterial sample (from step 3d) in 49 volumes of 1x ice-cold PBS to a final volume of 1 mL (i.e., 20 μL of cell sample in 980 μL of 1× PBS) in a 2 mL LoBind Eppendorf tube.
    ***Note:*** Keep the tube on ice.
  c. Load the input tube containing the bacterial cells on the FACS. Adjust the flow rate to reach ∼2,000 events per second.
    ***Note:*** If needed, the sample may be further diluted in PBS.
  d. Set gates to discriminate between intact *Bacteroides* cells and background noise (Figure 3).
    ***Note:*** The sort settings provided below may serve as a starting point, but the optimal photomultiplier tube (PMT) voltages for forward scatter (FSC) and side scatter (SSC) need to be determined empirically for each sample type and instrument. To sort *B*. *thetaiotaomicron* depending on cell size,^1^ we selected a linear FSC scale as to better resolve the variation in cell length. Neutral density filters are usually used to reduce the intensity of wavelengths in mammalian flow cytometry-based applications; however, since bacteria are small particles that evoke a weak light scatter, typically no neutral density filters are needed, unless fluorescence intensity reaches saturation.

**Table:** 

Parameter	Type	Axis	Voltage [V]
FSC	Area, height, width	Linear	600
SSC	Area, height, width	Logarithmic	500
FITC	Area, height, width	Logarithmic	879
5. Ensure accurate sorting of cells in individual wells of a 96-well plate.
  a. Seal an empty 96-well plate with a Microseal ‘B’ plate seal.
  b. Place the sealed plate in the Automatic Cell Deposition Unit (ACDU) (Figure 4A).

**Figure 4:**
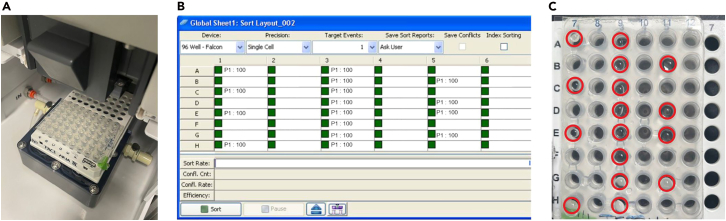
Sorting accuracy (A) The sealed plate is placed on the ACDU with the well at position A1 oriented to the top left corner. (B) Screenshot of the plate layout in the DIVA FACS software. The gated population is “P1“, as defined in Figure 3 and 100 cells are sorted per well in “single cell” precision mode. (C) Sorting accuracy is judged from visible droplet formation on top of the pre-selected wells. Red circles indicate the position of the droplets according to the layout in panel B.
  c. Install the splash shield below the sort aspirator.
  d. With only the left stream on, adjust the position of the ACDU to ensure the droplet is sorted into the A1 well of the plate.
  e. Sort bacterial cells (P1 in Figure 3B) onto the sealed plate, as outlined in Figure 4B.

***Note:*** Using a sealed plate facilitates the visualization of the sorted droplets (Figure 4C). We recommend confirming accurate sorting three times in this way, prior to sort-collecting the bacterial samples for sequencing.

***Note:*** Once the gating strategy is defined, the *Bacteroides* cells can be sorted into the prepared 96-well plate (step 2). For a first trial, we recommend to sort cells in decreasing numbers (e.g., 100, 10, 1; see Figure 5) in lane 3 of the plate (to avoid evaporation that can occur at the edges). This way, the sensitivity and efficiency of the protocol can be evaluated. When sorting single cells, it is advisable to leave a few randomly scattered wells on the plate deliberately empty, serving as intrinsic controls to evaluate the risk of spill-over and cross-contamination. In the case that the positive control does not display a characteristic profile on the bioanalyzer, see troubleshooting point 1, and if the negative control shows a contamination, see troubleshooting point 2. If the protocol will yield cDNA libraries for 100 and for 10 cells, but not for single cells, the lysis conditions may be sub-optimal. In such a case, see troubleshooting point 3.

**CRITICAL:** Upon harvest, sort the cells as soon as possible and directly into lysis buffer in order to minimize transcriptional changes in the sample and avoid contamination.

**Figure 6:**
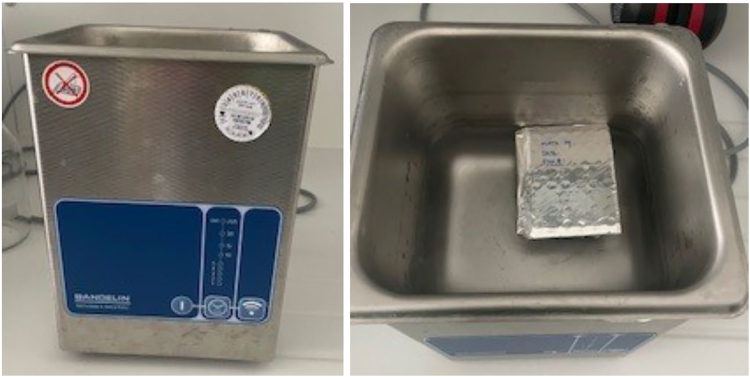
Sonicator used to lyse sorted *B*. *thetaiotaomicron* cells Left: Sonorex Digitec DT 52, Bendelin. Right: sealed plate deposited in 5 mL of water in the sonicator bath.
  h. Immediately after sonication, spin down the plate again, and place it on ice.
  i. Store the plate at −80°C for later processing.
    ***Note:*** In our experience, sonication is sufficient for the disruption of *Bacteroides* cells. However, cells from other bacterial genera may require more rigorous treatment for lysis. Specifically, *Enterobacteriaceae* are typically subjected to additional lysozyme treatment,^10^^,^^19^ whereas Gram-positive cells may necessitate supplementary mechanical disruption. We observed in previous experiments that enzymes employed for bacterial cell lysis can impede downstream reactions in the MATQ-seq protocol,^1^ leading us to omit enzymatic lysis in our protocol. In fact, this modification constitutes a significant deviation from preceding MATQ-seq-based single-bacterium RNA-seq protocols.^25^^,^^26^ See troubleshooting point 3.

6. Sort cells into the prepared plate (step 2).
  a. Dilute the cell sample in 49 volumes of 1× ice-cold PBS to a final volume of 1 mL (i.e., 20 μL of the cell sample in 980 μL of 1× PBS).
  b. Load the input tube containing the bacterial cells onto the FACS.
  c. Adjust the flow rate to reach ∼2,000 events per second.
    ***Note:*** If needed, the sample may be further diluted in PBS.
  d. Sort cells into the 96-well plate, using the single-cell purity mode.
    ***Note:*** Sorting a plate full of single bacteria only takes a few seconds. If sorting more cells (e.g. 100 cells) per well, the sorting can take up to a few minutes.
  e. Immediately after sorting, seal the plate with a Microseal ‘F’ plate seal.
    ***Note:*** While Microseal ‘B’ (see above: steps 2d, 5a) is clear and remains adhesive at −40°C, Microseal ‘F’ is an aluminized, pierceable, and peelable foil ideal for cold storage down to −70°C and thermal cycling.
  f. Spin the plate down for 5 s, using a mini plate spinner centrifuge.
  g. Place the plate in a sonicator bath (Figure 6) and sonicate for 20 s at 35 kHz.

**Figure 5:**
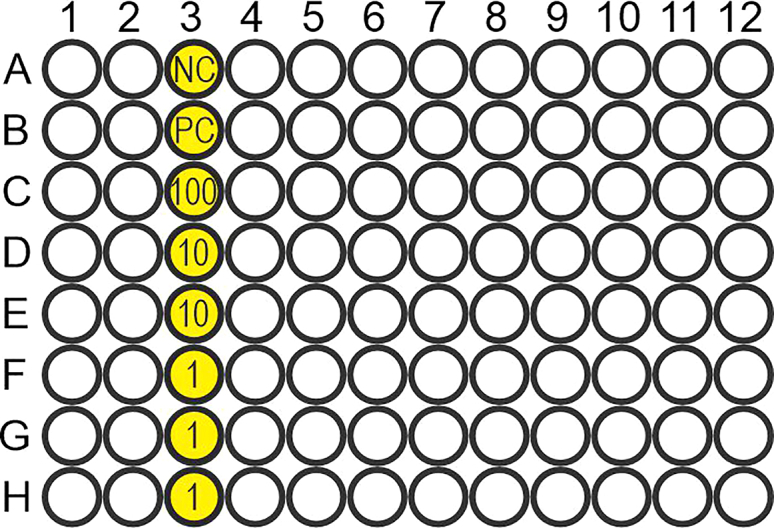
Schematic of the 96-well plate used for a pilot experiment Cells are sorted in lane 3 pre-filled with the reagents described in step 2. NC = negative control (a well containing only the reagents, but no sorted cell); PC = positive control (0.5 μL of 100 pg/μL total RNA extracted from *B. thetaiotaomicron*). 100, 10, and 1 stand for the number of sorted bacteria per well.

### MATQ-seq for single *B*. *thetaiotaomicron* cells

**Timing: ∼9 h (manual) or ∼5 h (when using a pipetting robot)**

**Figure 7:**
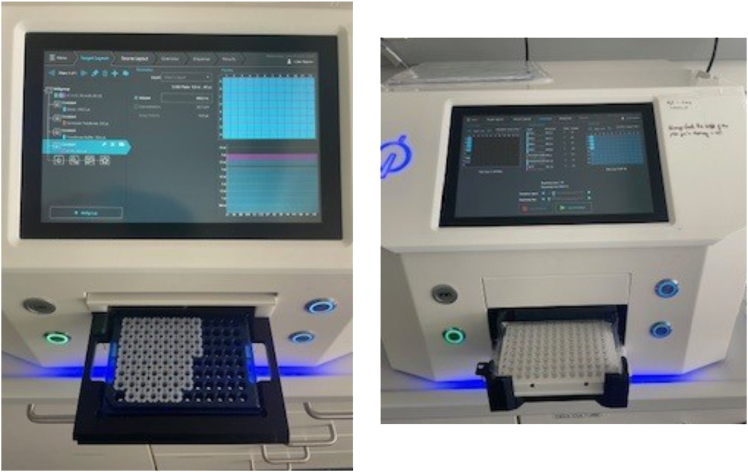
Liquid handling robot Left: I.DOT with the source plate open. In each individual well of the source plate, an enzyme or buffer has to be manually dispensed. From the source plate, reagents are then automatically dispensed into the target plate. To this end, the I.DOT calculates the exact pipetting volume depending on the number of selected target wells. Right: I.DOT with the target plate deposited.

This section describes the performance of the MATQ-seq protocol that converts RNA from single bacterial cells into first-strand cDNA, followed by second-strand synthesis and PCR amplification. The below indications refer to a single reaction. Volumes would thus need to be multiplied by the number of reactions needed for each experiment, plus an extra three reactions as a buffer. If available, pipetting can be automated using a liquid handling robot such as the I.DOT (Dispendix) (Figure 7).

**CRITICAL:** We recommend using separate thermocyclers for the reverse transcription and the PCR step to avoid cross-contamination.

7. Prepare the primer mix.
  a. Heat the lid of the thermocycler to 105°C.
  b. Defrost the 96-well plate that had been stored at −80°C (see step 6i).
  c. Defrost an aliquot of total RNA to use as a positive control (concentration 100 pg/μL) (see section “Preparation of positive control RNA samples”).
  d. While the plate is defrosting, manually prepare the primer mix in a 1.5 mL Eppendorf tube as follows:

**Table:** 

Reagent	Amount	Final concentration
GAT27 poly(dT) (100 μM)	0.12 μL	1.5 μM
GAT27 5N3G (100 μM)	0.4 μL	0.5 nM
GAT27 5N3T (100 μM)	0.4 μL	0.5 nM
Nuclease-free water	7.08 μL	N/A
    ***Note:*** The GAT27-MALBAC primer concentrations may be adjusted if a strong signal derived from primer dimers is seen on the Bioanalyzer (see troubleshooting point 4), but we generally recommend not to reduce the above concentrations by more than 10 folds.
  e. On a Combispin instrument, vortex the tube at low to medium speed for 5 s and spin it down for 5 s at 21°C.
8. Add each 0.5 μL of nuclease-free water (negative control) or 0.5 μL of 100 pg/μL pure RNA (positive control) to the specified wells.
9. “Pre-reverse transcription” denaturation
  a. Prepare the “pre-reverse transcription” master mix in a 1.5 mL Eppendorf tube:

**Table:** 

Reagent	Amount	Final concentration
DTT (0.1 M)	0.05 μL	1.5 mM
Primer mix (step 7d)	0.4 μL	N/A
dNTP (10 mM)	0.12 μL	1.4 mM
  b. On a Combispin instrument, vortex the tube at low to medium speed for 5 s and spin it down for 5 s at 21°C.
  c. Add 0.57 μL of the “pre-reverse transcription” mix to each well of the 96-well plate.
    ***Note:*** Pipetting tiny volumes by hand can introduce variability. It is therefore important to frequently calibrate your pipets. If variability remains high, use more volume of enzymes and buffers at lower concentrations or, if available, a pipetting robot.
  d. Seal the plate with a Microseal ‘B’ plate seal.
  e. Vortex the plate using a plate adaptor (speed 4, 10 s).
  f. Spin the plate down for 5 s, using a mini plate spinner centrifuge.
  g. Put the plate on a thermocycler and incubate for 3 min at 72°C.
  h. Immediately thereafter, put the plate on ice and let sit for at least 1 min.
10. Reverse transcription (RT).
  a. Prepare the RT master mix in a 1.5 mL Eppendorf tube:

**Table:** 

Reagent	Amount	Final concentration
SSIV buffer (5×)	0.8 μL	∼0.7x
DTT (0.1 M)	0.2 μL	3.5 mM
RNase inhibitor (40 U/μL)	0.1 μL	4 U
Superscript IV (200 U/μL)	0.15 μL	30 U
Nuclease-free water	1.15 μL	N/A
  b. On a Combispin instrument, vortex the tube at low to medium speed for 5 s and spin it down for 5 s at 21°C.
  c. Add 2.4 μL of the RT mix to each well of the 96-well plate.
  d. Seal the plate with a new Microseal ‘B’ plate seal.
  e. Vortex the plate using a plate adaptor (speed 4, 10 s).
  f. Spin the plate down for 5 s, using a mini plate spinner centrifuge.
  g. Put the plate on a thermocycler with the following settings:

**Table:** 

Steps	Temperature	Time	Cycles
Reverse transcription (ramping temperature)	8°C	12 s	11
	15°C	45 s	
	20°C	45 s	
	30°C	30 s	
	42°C	2 min	
Extension	50°C	3 min	–
Final extension	50°C	15 min	1
Hold	4°C	∞
  h. Spin the plate down for 5 s using a mini plate spinner centrifuge before proceeding to the next step.
    ***Note:*** Perform the subsequent steps at 21°C.
11. Digestion of residual DNA oligonucleotides.
  a. Incubate the plate for 1 min at 50°C.
  b. Add 0.2 μL of T4 DNA polymerase (3 U/μL) to each well (0.6 U/μL final concentration).
    ***Note:*** Here, the exonuclease activity of T4 DNA polymerase digests the primers. In the absence of dNTPs, the enzyme exhibits no polymerization activity.
  c. Seal the plate with a new Microseal ‘B’ plate seal.
  d. Vortex the plate using a plate adaptor (speed 4, 10 s).
  e. Spin the plate down for 5 s, using a mini plate spinner centrifuge.
  f. Incubate the plate as follows:

**Table:** 

Steps	Temperature	Time	Cycles
Digestion	37°C	40 min	1
Inactivation	75°C	20 min	1
Digestion	37°C	40 min	1
Inactivation	80°C	20 min	1
Hold	4°C	∞	–
    ***Note:*** Two sequential digestion/inactivation steps are performed to ensure complete and uniform 3′ end processing by T4 DNA polymerase. The partial heat-inactivation at 75°C will limit over-digestion, whereas the final heat-inactivation at 80°C will fully inactivate the enzyme.
  g. Spin down the plate for 5 s using a mini plate spinner, before proceeding to the next step.
12. RNA digestion.
  a. Prepare the RNA digestion master mix in a 1.5 mL Eppendorf tube as shown below:

**Table:** 

Reagent	Amount	Final concentration
RNase H (5 U/μL)	0.1 μL	0.5 U
RNase If (50 U/μL)	0.1 μL	5 U
    ***Note:*** For this step, we recommend preparing the mix for five extra reactions. This is because the enzyme solutions are very viscous and consequently, small volumes are difficult to pipet accurately.
  b. On a Combispin instrument, vortex the tube at low to medium speed for 5 s and spin it down for 5 s at 21°C.
  c. Add 0.2 μL of the RNA digestion mix to each well.
  d. Seal the plate with a new Microseal ‘B’ plate seal.
  e. Vortex the plate using a plate adaptor (speed 4, 10 s).
  f. Spin the plate down for 5 s using a mini plate spinner centrifuge.
  g. Incubate as follows:

**Table:** 

Steps	Temperature	Time	Cycles
Digestion	37°C	15 min	1
Inactivation	72°C	15 min	1
Hold	4°C	∞	–
  h. Spin the plate down for 5 s using a mini plate spinner centrifuge, before proceeding to the next step.
13. Poly(C)-tailing of cDNA.
  a. Prepare the tailing master mix in a 1.5 mL LoBind Eppendorf tube as follows:

**Table:** 

Reagent	Amount	Final concentration
Terminal transferase buffer (10×)	0.4 μL	0.4×
dCTP (100 mM)	0.4 μL	4 mM
Terminal transferase (20 U/μL)	0.1 μL	2 U
Nuclease-free water	3.13 μL	N/A
  b. On a Combispin instrument, vortex the tube at low to medium speed for 5 s and spin it down for 5 s at 21°C.
  c. Add 4.03 μL of the tailing master mix to each well.
  d. Seal the plate with a new Microseal ‘B’ plate seal.
  e. Vortex the plate using a plate adaptor (speed 4, 10 s).
  f. Spin the plate down, for 5 s using a mini plate spinner centrifuge.
  g. Incubate as follows:

**Table:** 

Steps	Temperature	Time	Cycles
Poly(C)-tailing	37°C	15 min	1
Inactivation	72°C	15 min	1
Hold	4°C	∞	–
  h. Spin the plate down for 5 s using a mini plate spinner centrifuge, before proceeding to the next step.
14. Second-strand cDNA synthesis.
  a. Prepare the second-strand cDNA synthesis master mix in a 1.5 mL Eppendorf tube as follows:

**Table:** 

Reagent	Amount	Final concentration
Thermopol buffer (10x)	1.5 μL	0.5x
dNTP (10mM)	1.25 μL	0.5 mM
GAT21 6N3G (100 μM)	0.125 μL	0.5 μM
Nuclease-free water	12.925 μL	N/A
  b. On a Combispin instrument, vortex the tube at low to medium speed for 5 s and spin it down for 5 s at 21°C.
  c. Add 15.8 μL of the second-strand cDNA synthesis master mix to each well.
  d. Seal the plate with a new Microseal ‘B’ plate seal.
  e. Vortex the plate using a plate adaptor (speed 4, 10 s).
  f. Spin the plate down, for 5 s using a mini plate spinner centrifuge.
  g. Incubate as follows:

**Table:** 

Temperature	Time
95°C	1 min
Cool down to 48°C	–
  h. Spin the plate down for 5 s using a mini plate spinner centrifuge, before proceeding to the next step.
  i. Add 0.4 μL of DeepVent(exo-) DNA polymerase to each well.
  j. Seal the plate with a new Microseal ‘B’ plate seal.
  k. Vortex the plate using a plate adaptor (speed 4, 10 s).
  l. Spin the plate down, for 5 s using a mini plate spinner centrifuge.
  m. Incubate as follows:

**Table:** 

Steps	Temperature	Time	Cycles
Reverse transcription and looping	48°C	20 s	11
	72°C	1 min	–
Final extension	72°C	2 min	1
Hold	4°C	∞	–
    ***Note:*** The total volume per well is 26.2 μL.
15. Dispense ¼ of each reaction (=6.55 μL) over a new 96-well plate.

***Note:*** This new plate will be used for the ensuing PCR amplification. The former plate can be sealed and stored at −20°C as a backup.

16. PCR amplification.
  a. Prepare the PCR master mix in a 5 mL Eppendorf tube as follows:

**Table:** 

Reagent	Volume	Final concentration
Thermopol buffer (10x)	3.25 μL	0.8x
dNTP (10 mM)	0.75 μL	0.18 mM
GAT27 PCR (100 μM)	0.2 μL	0.5 μM
DeepVent (exo-) (2 U/μL)	0.75 μL	1.5 U
Nuclease-free water	28.5 μL	N/A
  b. On a Combispin instrument, vortex the tube at low to medium speed for 5 s and spin it down for 5 s at 21°C.
  c. Add 33.45 μL of the PCR master mix to each well.
  d. Seal the plate with a new Microseal ‘B’ plate seal.
  e. Vortex the plate using a plate adaptor (speed 4, 10 s).
  f. Spin the plate down, for 5 s using a mini plate spinner centrifuge.
  g. Incubate as follows:

**Table:** 

Step	Temperature	Time	Cycles
Initial denaturation	95°C	30 s	1
Denaturation	95°C	15 s	25
Annealing	62°C	20 s	
Extension	72°C	2 min	
Final extension	72°C	5 min	1
Hold	4°C	∞	–
    **Pause point:** At this point, the plate can be stored at −20°C for later clean-up. However, do not store the unpurified samples at this stage for more than 2 days (see troubleshooting point 3).
17. PCR reaction clean-up.
  a. To each PCR reaction sample (40 μL total volume), add 40 μL of AMPure XP beads (1:1 ratio [v/v]).
  b. Mix well by gentle pipetting.
  c. Incubate the samples for 10 min at 21°C.
  d. Put the plate onto a magnetic stand for the beads to pellet.
    **CRITICAL:** Wait for the solution to clear before proceeding to the next step (typically takes ∼2 min).
  e. Carefully take off and discard the supernatant with the plate still on the magnetic stand.
    **CRITICAL:** Avoid touching the beads with the pipet tip.
  f. Keep the plate on the magnetic stand and wash the beads twice with each 180 μL of freshly prepared 80% ethanol in water.
  g. In between, incubate for 30 s.
  h. Discard the supernatant.
  i. After the second wash, air-dry the beads for 5-10 min with open lid.
    **CRITICAL:** The ethanol should completely evaporate, but over-drying should be avoided as it hampers elution.
    ***Note:*** The dried beads should still appear glossy, whereas over-dried beads would form cracks.
  j. Remove the plate from the magnetic stand.
  k. Add each 15 μL of nuclease-free water per well.
    ***Note:*** Ensure proper resuspension of the beads in the water.
  l. Incubate for 2 min at 21°C.
  m. Place the plate back onto the magnetic stand.
  n. Wait for ∼2 min, until the beads have pelleted.
  o. Transfer each 13 μL of the eluate into a new 96-well plate and discard the former plate (containing the magnetic beads).
18. Quality assessment.
  a. Measure the cDNA concentration in each sample using a Qubit 1x dsDNA high-sensitivity kit according to the manufacturer’s recommendation (Qubit™ 1X dsDNA HS Assay Kits User Guide (MAN0017455 Rev C.0)). Refer to the expected outcome section of the protocol for anticipated results.
  b. Select up to 11 individual samples (that is how many fit on a chip) covering the full spectrum of measured cDNA concentrations (minimum, intermediate, and maximum concentrations) to run on a Bioanalyzer.
  c. Dilute those samples to a concentration of each 1 ng/μL with nuclease-free water before loading and running them on a high-sensitivity DNA chip according to the manufacturer’s recommendations (Agilent High Sensitivity DNA Kit Quick Start Guide) (Figure 8).

**Figure 8:**
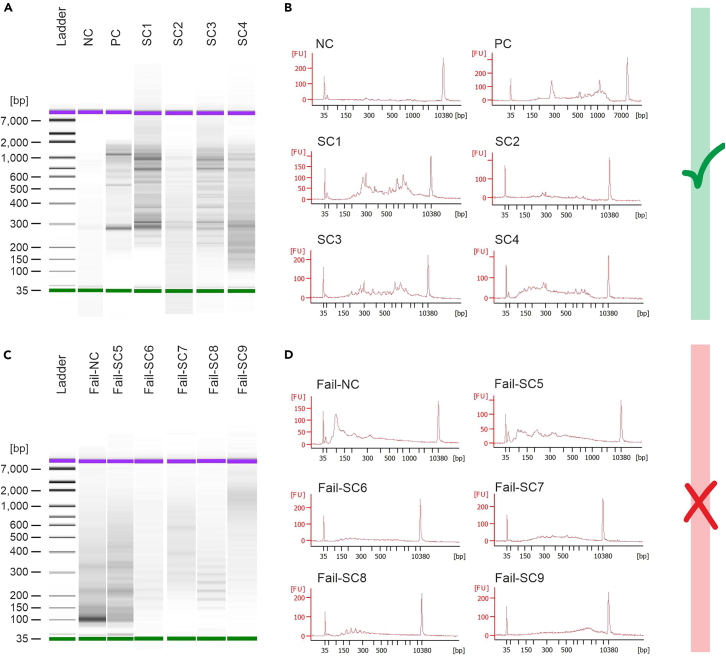
Representative Bioanalyzer plots after MATQ-seq-based reverse transcription (A) Gel-like image for a negative control (NC), a positive control (PC), and 4 successful single bacterial cells (SC1-4). Each lane represents one sample. The green and purple lines denote the lower (35 bp) and upper (10,380 bp) marker, respectively. Successful attempts should display peaks at positions comparable to those of the PC, namely between 300 and 1,000 bp. SC2 is a borderline case, but faint signals at the respective heights were detected and the sample was eventually sequenced successfully. (B) Electropherograms of the samples in panel (A). FU, fluorescence intensity unit. On the x-axis, the size of the DNA fragments is displayed. (C and D) Gel-like image (C) and electropherograms (D) of a contaminated negative control (Fail-NC) and failed attempts of 4 single bacterial cells (Fail-SC5-9). For Fail-SC5-9, the representative profile of the PC (seen in panels a and b, with peaks at 300-1,000 bp) was not recovered.

**Pause point:** At this point, the plate can be stored at −20°C for several months until further processing.

### Preparation of Illumina sequencing libraries

**Timing: ∼7 h (manual) or ∼5 h (when using a pipetting robot)**

From the amplified cDNAs of the previous step (step 17o), sequencing libraries will be prepared using the Nextera XT kit (Illumina). In our experience, the volumes recommended by the manufacturer can be scaled down to 1/4^th^ and the below volumes reflect this downscaling. (With the I.DOT liquid handling robot, we have successfully downscaled the reaction volumes to even 1/10^th^ of the recommended volumes).

19. Prepare reagents.
  a. In a new 96-well plate, dilute the cDNA samples to a concentration of 0.4 ng/μL using nuclease-free water.
  b. Thaw the Nextera reagents (Amplicon Tagment Mix [ATM], Tagment DNA [TD] buffer) on ice.
  c. Thaw the Unique Dual Index (UDI) primers.
  d. Warm up the Neutralize Tagment (NT) buffer to 21°C and dissolve potential precipitates by vortexing.
20. Mix reagents for library tagmentation.
  a. In individual wells of a new 96-well plate, mix 1.25 μL of the 0.4 ng/μL-concentrated cDNA (step 19a) with 2.5 μL of TD buffer.
  b. Add 1.25 μL of ATM.
  c. Mix the samples well, either by pipetting or brief vortexing of the sealed plate on a plate adaptor.
  d. Spin the plate down for 5 s using a mini plate spinner centrifuge.
21. Tagmentation.
  a. Incubate the plate for 10 min at 55°C.
    **CRITICAL:** Do not incubate for more than 10 min, as this may result in over-tagmentation of the library (see troubleshooting point 5).
  b. Cool down the samples to 10°C and immediately add each 1.25 μL of NT buffer.
  c. Mix the samples as above (step 20c).
  d. Spin the plate down for 5 s on a mini plate spinner centrifuge.
  e. Incubate at 21°C for 5 min.
22. Amplify libraries.
  a. Add 3.75 μL of the Nextera PCR master mix (NTM) to each well.
  b. Add 2.5 μL of UDI primers to each sample.
    **CRITICAL:** Keep track of the indices used for each sample.
  c. Mix the samples by gently vortexing the plate on a plate adaptor (speed 2, 10 s).
  d. Spin the plate down for 5 s on a mini plate spinner centrifuge.
  e. Run the following PCR program to amplify the libraries:

**Table:** 

Steps	Temperature	Time	Cycles
Extension of adapters	72°C	3 min	1
Denaturation	95°C	30 s	12––
Annealing	55°C	15 s	
Extension	72°C	20 s	
Final extension	72°C	5 min	1
Hold	10°C	∞	–
23. Library clean-up.
  a. Purify the libraries using a 1:1 [v/v] ratio of beads to sample. In the present case, add 12.5 μL of AMPure XP beads per sample. Follow the instructions as described in step 17a-j.
  b. Elute the libraries in each 13.1 μL of nuclease-free water.
  c. Transfer 11 μL of each eluate to the wells of a new 96-well plate.
24. Quality control.
  a. Measure the cDNA concentration of each sample using a Qubit 1X dsDNA high-sensitivity kit following the manufacturer’s instructions (Qubit™ 1X dsDNA HS Assay Kits User Guide (MAN0017455 Rev C.0)).
    ***Note:*** Usually, the concentrations should range between 2 and 10 ng/μL. Refer to the expected outcome section (see below) for anticipated results.
  b. Select samples covering the full range of measured concentrations.
  c. Dilute the selected cDNA samples to reach a concentration of 1 ng/μL with nuclease-free water.
  d. Run the samples on a Bioanalyzer (high-sensitivity DNA chip) following the manufacturer’s instructions (Agilent High Sensitivity DNA Kit Quick Start Guide) (Figure 9).
    **Pause point:** At this point, the plate can be stored at −20°C for several months.

**Figure 9:**
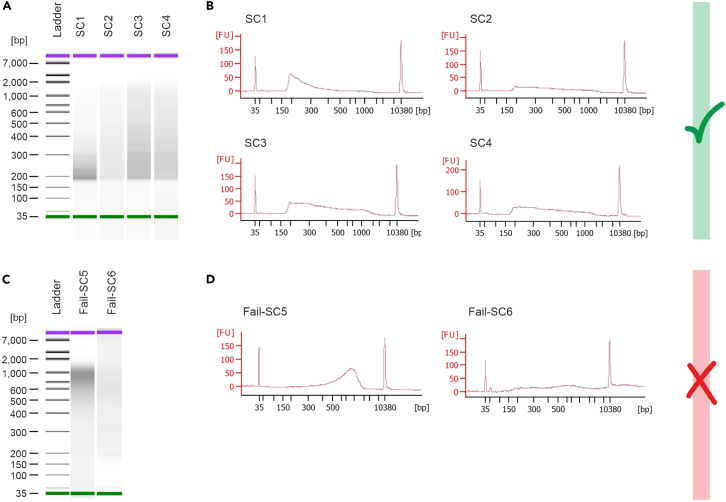
Representative Bioanalyzer plots after cDNA library construction via Nextera XT Gel-like images (A and C) and electropherograms (B and D) of Nextera XT libraries obtained from single *B*. *thetaiotaomicron* cells. Panels A and B show successful libraries, while panels C and D depict failed experiments. For example, the tagmentation of the library “Fail-SC5” was unsuccessful, with the library displaying a peak at ∼800 bp. In the case of “Fail-SC6”, no library was recovered at all. See legend to Figure 8 for details.

### Ribosomal cDNA depletion via DASH

**Timing: 2 days**

In bacteria, the 5S, 16S, and 23S rRNAs are the most abundant cellular transcripts. However, due to their low informative value, rRNAs are typically depleted from cDNA libraries prior to sequencing. In the present protocol, the removal of ribosomal sequences is achieved via the sensitive depletion of abundant sequences by hybridization (DASH) protocol that programs Cas9 nuclease to cleave ribosomal cDNA fragments.^27^ However, before performing DASH (step 26), the corresponding single-guide RNAs (sgRNAs) need to be synthesized (step 25). Established oligonucleotides used previously to *in vitro*-transcribe sgRNAs for *B*. *thetaiotaomicron* ribosomal sequences^1^^,^^27^ are contained in Table S1.

25. Prepare the sgRNA library.
  a. Using nuclease-free water, dilute the oligonucleotide pool and fill-in reaction oligonucleotides to each 10 μM.
    ***Note:*** When purchased from Integrated DNA Technologies, Inc. (IDT), by default the oligonucleotide pools are delivered in a final concentration of 50 μM. To dilute them to 10 μM, add 10 μL of the oligonucleotide pool to 40 μL of nuclease-free water in a 1.5 mL LoBind Eppendorf tube. The concentration of the fill-in reaction primer stock has been adjusted to 100 μM. To dilute it to 10 μM, 5 μL of the fill-in primers are mixed with 45 μL of nuclease-free water in a 1.5 mL LoBind Eppendorf tube.
  b. Generate the double-stranded DNA (dsDNA) template for sgRNA *in*-*vitro* transcription.
    i. In a 1.5 mL tube, prepare the following mix on ice:

**Table:** 

Reagent	Amount	Final concentration
Oligonucleotide pool (10 μM)	5 μL	1 μM
Fill-in oligonucleotides (10 μM)	10 μL	2 μM
KAPA HiFi HotStart Ready mix (2×)	25 μL	1×
Nuclease-free water	10 μL	N/A
    ii. Mix gently by pipetting up and down.
    iii. Spin down the tube in a tabletop centrifuge.
    iv. Incubate in a thermocycler (with the lid heating set to 105°C):

**Table:** 

Steps	Temperature	Time	Cycle
Denaturation	95°C	3 min	1
Annealing	60°C	20 s	1
Extension	72°C	1 min	1
Cooling	4°C	∞	Hold
      ***Note:*** To increase the yield, two reactions can be pipetted in parallel and pooled after the column PCR clean-up.
    v. Column-purify the PCR product with the Zymo Oligo Clean & Concentrator kit following the manufacturer’s instructions (d4060_d4061_oligo_clean_concentrator.pdf).
    vi. Elute the sample in 15 μL of nuclease-free water.
    vii. Measure the concentration of dsDNA on a NanoDrop.
    viii. Assess the quality of the sample with a high-sensitivity DNA Bioanalyzer chip (Figure 10), according the manufacturer’s instructions (Agilent High Sensitivity DNA Kit Quick Start Guide).
      **Pause point:** The dsDNA template can be stored at −20°C for several months.

**Figure 10:**
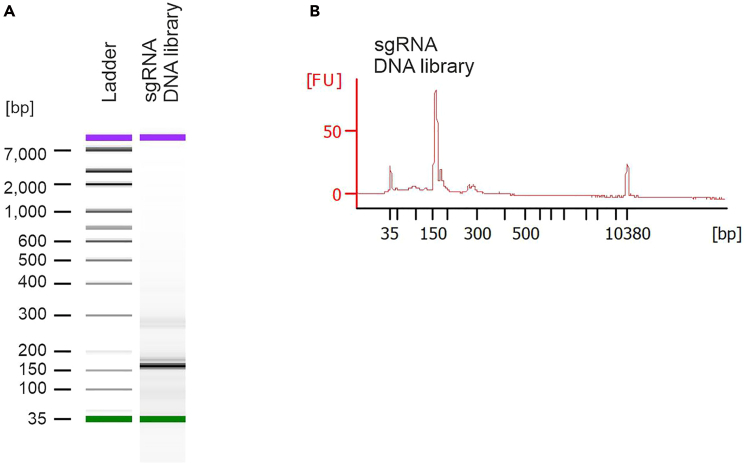
Representative Bioanalyzer plots of single-guide RNAs Gel-like image (A) and electropherogram (B) of sgRNA dsDNA obtained from step 25. See legend to Figure 8 for details.
  c. Synthesize sgRNAs by *in*-*vitro* transcription.
    i. Dilute the dsDNA template to 150 ng/μL in nuclease-free water.
    ii. Mix the following reagents from the MEGAshortscript kit (ThermoFischer Scientific) at 21°C and add the dsDNA template to a final concentration of 300 ng in 20 μL:

**Table:** 

Reagent	Amount	Final concentration
T7 10× reaction buffer	2 μL	1x
NTP mix (mix 2 μL of each rNTP provided in the kit)	8 μL	10 mM each (40 mM total)
dsDNA template (150 ng/μL)	2 μL	15 ng/µL
T7 enzyme mix	2 μL	N/A
Nuclease-free water	6 μL	N/A
    iii. Mix gently by pipetting up and down.
    iv. Spin down the tube.
    v. Incubate on a thermocycler.
      **CRITICAL:** The lid heating should now be turned off.

**Table:** 

Steps	Temperature	Time	Cycles
Incubation	37°C	4 h	1
Cooling	4°C	∞	Hold
    vi. Take the tube out of the thermocycler and spin it down.
    vii. Add 1 μL of TURBO DNase from the MEGAshortscript kit.
    viii. Mix gently by pipetting up and down, then spin down.
    ix. Incubate in the thermocycler (with the lid heating still turned off):

**Table:** 

Steps	Temperature	Time	Cycles
Incubation	37°C	30 min	1
Cooling	4°C	∞	Hold
    x. Perform the RNA clean-up using the Monarch Spin RNA Cleanup kit (500 μg), according to the manufacturer’s recommendations (Monarch® Spin RNA Cleanup Kit Protocol | NEB).
    xi. Elute in 50 μL of nuclease-free water.
    xii. Assess the quality of the resulting sgRNAs by determining the RNA concentration of the sample on a NanoDrop.
    xiii. Check the size of the sgRNA pool using a Bioanalyzer Pico kit, following the manufacturer’s recommendations (RNA 6000 Pico Kit for 2100 Bioanalyzer Systems Kit Guide).
      ***Note:*** Expected is a single peak of between 150 and 160 bp.
      **Pause point:** The sgRNA pool can be stored at −80°C for several months.
26. Ribo-depletion from single-bacterium cDNA samples.
  a. Calculate the molarity of the cDNA samples (steps 23c and 24a,d), and the sgRNA (step 25).
  b. Pool up to 12 cDNA libraries equimolarly in PCR strip tubes.
    ***Note:*** From step 24, the average library size (Bioanalyzer traces; step 24d) and concentration (Qubit measurements; step 24a) of the cDNA samples are known and can here be used to calculate their molarity. Based on this, all libraries should be diluted to match the molarity of the lowest concentrated sample (in our hands, the minimal molarity observed here was 2.1 fmol). The thus adjusted samples (maximally 12) will then be pooled equimolarly.
    ***Note:*** The spreadsheet in Table S2 serves as a template to calculate molarities and to pool the samples.
  c. Calculate the required volumes to reach the intended ratio of cDNA:Cas9:sgRNA.
    ***Note:*** We typically work with a ratio of 1:1,000:2,000 (cDNA:Cas9:sgRNA).
  d. Prepare the sgRNA-Cas9 ribonucleoprotein (RNP) complex.
    i. Denature the calculated volume of the sgRNA pool from step 25 for 3 min at 95°C, followed by 5 min at 4°C.
    ii. Add the calculated volume of Cas9 to the denatured sgRNA pool.
    iii. Incubate for 15 min at 37°C.
  e. Add the sgRNA-Cas9 RNP complex to the PCR tube containing the pooled cDNA libraries (step 26b).
  f. For Cas9-mediated digestion of ribosomal cDNAs, incubate the sgRNA-Cas9 RNP complex with the cDNAs for 2 h at 37°C.
  g. Inactivation of Cas9.
    i. Add 1 μL of proteinase K.
    ii. Incubate in a thermocycler as follows:

**Table:** 

Step	Temperature	Time	Cycle
Digestion of Cas9	37°C	15 min	1
Heat-inactivation of proteinase K	95°C	15 min	1
      **CRITICAL:** Immediately after the heat-inactivation, proceed to the AMPure bead clean-up.
  h. Perform AMPure bead clean-up (as described in step 17) at a 1:1 [v/v] ratio of sample:beads and elute in 15 μL of nuclease-free water.
  i. Measure the sample concentrations on a Qubit Flex fluorometer using the 1x dsDNA high-sensitivity kit following the manufacturer’s instructions (Qubit™ 1X dsDNA HS Assay Kits User Guide (MAN0017455 Rev C.0)).
  j. Adjust the library concentrations to 0.5 ng/μL with nuclease-free water.
  k. Perform the last PCR indexing reaction.
    i. Mix the following reagents:

**Table:** 

Reagent	Amount	Final concentration
cDNA library (0.5 ng/μL)	6.25 μL	0.25 ng/μL
Nextera PCR master mix (NTM)	3.75 μL	N/A
DASH amplification primer (forward)	1.25 μL	1 μM
DASH amplification primer (reverse)	1.25 μL	1 μM
    ii. Mix the sample by gently pipetting up and down.
    iii. Spin down for 5 s.
    iv. Run the following PCR program to amplify the ribosomal cDNA-depleted library:

**Table:** 

Step	Temperature	Time	Cycles
Extension of adapters	72°C	3 min	1
Denaturation	95°C	30 s	12
Annealing	55°C	15 s	–
Extension	72°C	20 s	–
Final extension	72°C	5 min	1
  l. Clean up the reaction using AMPure beads (as described in step 17) at a 1:1 [v/v] ratio of sample:beads and elute in 15 μL of nuclease-free water.
  m. Measure the concentration on the Qubit Flex using the 1x dsDNA high-sensitivity kit following the manufacturer’s instructions (Qubit™ 1X dsDNA HS Assay Kits User Guide (MAN0017455 Rev C.0)).
  n. Dilute the sample to 1 ng/μL.
  o. Check the size of the DASHed cDNA library using a Bioanalyzer high-sensitivity DNA analysis kit, following the manufacturer’s recommendations (Agilent High Sensitivity DNA Kit Quick Start Guide) (Figure 11).

**Figure 11:**
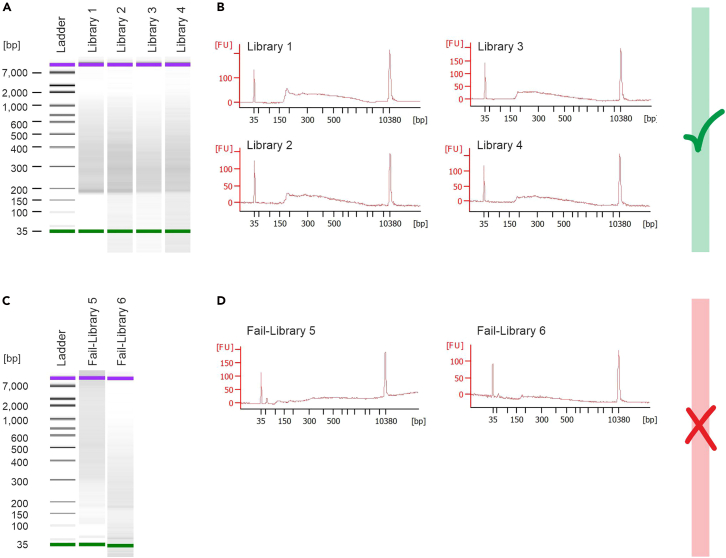
Representative Bioanalyzer plots of final sequencing libraries Representative Bioanalyzer gel-like images (A and C) and electropherograms (B and D) of rRNA-depleted cDNA libraries. Panels A and B show successful libraries. Panels C and D show two examples where no library could be recovered, suggesting either a failed PCR amplification or a problem during the DASH procedure. See legend to Figure 8 for details.

### Sequencing and data analysis

**Timing: ∼2 days(depending on the sequencing platform)**

27. Sequencing.
  a. Measure the concentrations of the obtained ribosomal cDNA-depleted libraries with a Qubit Flex and determine the average library sizes with a Bioanalyzer.
  b. Calculate library molarity from the library concentration and average library size using the following equation:
    $$nM=\frac{library\;concentration\left(\frac{ng}{ul}\right)}{66O\left(\frac{g}{mol}\right)\;*\;average\;library\;size\;\left(bp\right)}*1,000,000$$
    where 660 g/mol represents the average weight of a DNA base pair.
  c. Adjust the concentration of all libraries to 5 nM using sequencing buffer (10 mM Tris-HCl, pH 8.5, 0.1% [v/v] Tween-20).
  d. Pool libraries equimolarly.
  e. Measure the concentration of the pool on a Qubit Flex, according to the manufacturer’s recommendations (Qubit™ 1X dsDNA HS Assay Kits User Guide (MAN0017455 Rev C.0)).
  f. Check the quality of the pool and the library size with a Bioanalyzer high-sensitivity DNA kit, according to the manufacturer’s recommendations (Agilent High Sensitivity DNA Kit Quick Start Guide).
  g. Sequence on an Illumina platform in single-end mode with 100 cycles.
    ***Note:*** We recommend a minimum sequencing depth of ∼10 million reads per single-bacterium library.
28. Read mapping.
  a. Download the *B. thetaiotaomicron* VPI-5482 reference genome and plasmid (NCBI RefSeq assembly GCF_000011065.1) as both fasta and gff files.
  b. Trim the obtained sequencing reads from the Nextera adapters and the MATQ-seq primers on both their 5′ and 3′ ends using BBDuk.^31^
  c. Assess the quality of the trimming with FASTQC.^33^
  d. Map the trimmed reads to the *B. thetaiotaomicron* VPI-5482 reference genome and plasmid using Bowtie 2.^32^
  e. Using featureCounts^34^ and gff files, quantify the mapped reads.

***Note:*** The script for this data analysis has been previously published^19^ and can be found on https://github.com/BarquistLab/MATQ-seq_2023, in the folder Bash_script.sh. The genome and gff files need to be changed to *B*. *thetaiotaomicron*.

## Expected outcomes

After cDNA synthesis using the MATQ-seq protocol, all libraries are measured using the Qubit Flex (step 18a). The concentration of the positive control should be between 5 and 25 ng/μL. The negative control’s concentration should be below 2 ng/μL. Libraries derived from single *B*. *thetaiotaomicron* bacteria usually have concentrations between 3 and 10 ng/μL at this point in the protocol.

When running the adjusted samples on a DNA high-sensitivity Bioanalyzer chip (step 18c), the traces of the negative and positive controls and those of the single-bacterium libraries should resemble the examples shown in Figure 8A and 8B. If not (Figure 8C and 8D), see troubleshooting points 1-4.

After cDNA library preparation using the Illumina Nextera XT kit (step 24a), the DNA concentration of single-bacterium samples should range between 2 and 10 ng/µL. Representative plots obtained after running the Nextera libraries on a Bioanalyzer high-sensitivity DNA kit are presented in Figure 9A and 9B. In the case that your cDNA libraries give rise to different trace patterns (Figure 9C and 9D), consult troubleshooting point 5.

The sgRNA library concentration should be ∼1 μg/μL on the NanoDrop (step 25b, vii). Figure 10 shows the expected representation of the dsDNA sgRNA library when analyzed with a Bioanalyzer high-sensitivity DNA kit (step 25b, viii).

After rRNA-derived cDNA depletion via DASH and the second PCR amplification, the DNA concentration of the single-bacterium library pools should range between 4 and 10 ng/μL (step 26m). When run on a Bioanalyzer (step 26o), rRNA-depleted, pooled libraries should resemble the ones shown in Figure 11A and 11B; otherwise (Figure 11C and 11D), see troubleshooting point 6.

**Figure 12:**
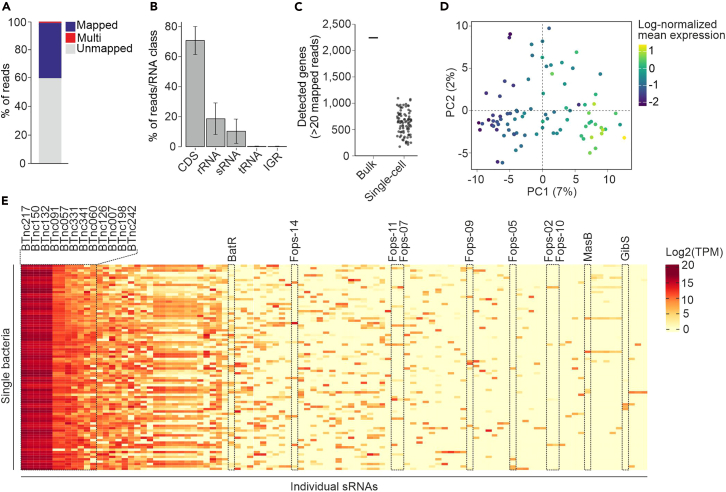
RNA-seq results of 89 single *B*. *thetaioatomicron* cells grown in TYG medium to mid-exponential phase (A) Mapping statistics averaged over 89 single bacteria. ‘Multi’ refers to multi-mapped reads, i.e., reads that aligned to more than one genomic locus. (B) RNA class distribution across the same 89 bacteria. Bar plots indicate the mean and error bars represent the standard deviation. CDS: coding sequence; rRNA: ribosomal RNA; sRNA: small RNA; tRNA: transfer RNA; IGR: intergenic region. (C) Number of detected genes in each of the 89 single cells or in a corresponding bulk RNA-seq dataset.^35^ (D) Principal component analysis plot of the 89 single-bacterium transcriptomes, colored based on the log-normalized mean expression of genes classified as variable between cells. Along principal component 1, bacteria showed a gradual increase in the expression of genes identified as highly variable (encoding ribosomal and cell structural proteins). (E) Log2 transcript per million (TPM) expression of sRNAs detected in at least one of the 89 single-bacterium libraries. Universally expressed and functionally characterized sRNAs^35^^,^^36^^,^^37^^,^^38^ are labeled.

Of 93 single *B*. *thetaiotaomicron* cells harvested at mid-exponential phase (OD_600_ = 2.0), 89 cDNA libraries were successfully obtained, resulting in an overall efficiency of 96%. This 4% dropout rate reflects sorting mistakes, i.e., the FACS instrument will “miss” a given well in ∼2-10% of cases.^1^ Sequencing data analysis showed that ∼40% of the reads mapped to the *B*. *thetaiotaomicron* VPI-5482 genome (Figure 12A). Most of the “junk” reads were poly(C) and poly(G) stretches and derived from poly(C)-tailing during second-strand synthesis. Of the *Bacteroides*-mapped reads, an average of 70% aligned to coding sequences and only 20% to rRNA loci (Figure 12B), reflecting efficient ribosomal cDNA depletion via DASH at the single-bacterium level. A median of 742 genes were detected per cell (cutoff for detection: >20 mapped reads) (Figure 12C). Analysis of transcriptional heterogeneity within the *B*. *thetaiotaomicron* population revealed that mRNAs for ribosomal and cell structural proteins were amongst the most variably detected transcripts. This suggests that the expression of these genes is a main driver of heterogeneity between individual *B*. *thetaiotaomicron* cells in mid-exponential phase (Figure 12D).

The protocol also readily captures small RNAs (sRNAs) (Figure 12B)—an important class of non-protein-encoding post-transcriptional regulators in the prokaryotic kingdom.^39^ In our dataset, ∼200 of the 350 annotated *B*. *thetaiotaomicron* sRNA candidates^36^ were detected across the 89 single bacteria (using the same cutoff as for coding transcripts). Next to a few universally present sRNAs, many others were seemingly heterogeneously expressed (Figure 12E). However, future studies should investigate whether these sRNAs are truly bistably expressed or if their presence/absence reflects stochastic loss or retention in individual cells.

## Limitations

MATQ-seq for bacteria is a highly sensitive, probe-independent single-bacterium RNA-seq method, but it is limited by low throughput (due to the physical isolation of bacterial cells typically only a few hundred individual reactions can be performed in parallel), a relatively high cost, especially when the sgRNA template library for DASH needs to be synthesized (2.9 € per cell), and a long, labor-intensive workflow (∼5 days). Alternative approaches to decipher phenotypic heterogeneity within bacterial populations have been developed, with other strengths and weaknesses, that can complement the present protocol. Combinatorial indexing-based single-cell RNA-seq techniques such as microSplit-seq^11^ and PETRI-seq^9^ assign RNA transcripts to cells using combinatorial barcoding. This bypasses the need for physical isolation of individual cells and yields a higher throughput (∼10,000 bacterial transcriptomes per experiment). Combinatorial indexing-based approaches have been successfully applied to various bacterial species grown both in planktonic state and in biofilms, demonstrating robust discrimination of cell-states across growth conditions and during phage infection.^40^ Besides, droplet-based techniques such as BacDrop,^13^ M3-seq,^12^ and smRandom-seq^14^ have also been adopted for single-bacterium RNA-seq. These approaches allow for a very high throughput (∼100,000 bacterial transcriptomes per experiment) and have primarily been used to study bacterial responses to antibiotic treatment. The downside of these alternative methods relates to their comparably high dropout rates (∼30%, as compared to 4% in MATQ-seq) and lower sensitivity (∼200 genes/cell, as compared to >700 genes/cell with the present protocol), typically preventing the detection of sRNAs. However, none of these methods have yet been applied to *Bacteroides* cells, precluding a direct comparison to our present protocol.

## Troubleshooting

### Problem 1

The positive control does not yield a cDNA library in the MATQ-seq section for single *B*. *thetaiotaomicron* cells (step 7-18).

### Potential solution

• Reassess quality and concentration of total extracted RNA on a NanoDrop. The A260/280 ratio should be >2. If this ratio is below 1.9, repeat the total RNA extraction.
• The RNA concentration of the positive control should be ∼100 pg/μL. To adjust the sample to such a low concentration, it is essential to perform serial dilutions.
• Use a fresh aliquot of MATQ-seq primers (step 7).

### Problem 2

The negative control gives rise to traces on the Bioanalyzer (section “MATQ-seq for single *B*. *thetaiotaomicron* cells”). This issue suggests a contamination and might be observed sporadically, especially when reagents are shared between users or when the protocol was paused and re-initiated after an extended time period.

### Potential solution

• Contaminations can e.g., occur during the plate preparation (step 2) and during the MATQ-seq protocol (steps 7-17). In both cases, ensure that you are working under a sterile PCR hood and thoroughly disinfect your work bench and spray it with RNase ZAP before pipetting.
• Contaminations can also occur during cell-sorting. Before using the FACS, ensure that the flow cell is clean. If not, the flow cell should be cleaned using the “Clean flow cell” option of the FACS. Alternatively, or in addition, the stream can be run at a high flow rate (e.g., 11) for a few minutes with water or FACS Clean solution to clean the tubing and resolve potential clogs. Also, work quickly when unsealing the plate for sorting and immediately reseal the plate containing the sorted cells.
• Make sure to aliquot all reagents, buffers, and enzymes when delivered and before using them for the first time. In doing so, if a contamination is suspected, a fresh aliquot can be used.
• Include nuclease-free water in every Bioanalyzer high-sensitivity run to exclude the contamination of the used water.

### Problem 3

cDNA libraries can be obtained for 100 and 10 bacteria, but not for a single bacterium (step 18).

### Potential solution

• This is likely due to an inefficient lysis of bacterial cells. In this case, we recommend to optimize the cell lysis conditions. The table below shows different lysis strategies that can be used to disrupt Gram-negative bacterial cells (see also ^26^). Additionally, quick freeze and thaw cycles can be performed to further improve lysis efficiency. To this end, place the 96-well plate containing the sorted bacteria on dry ice for a few seconds before removing it and repeat this a few times.

**Table:** 

	Starting concentration	Final concentration	Volume (without lysozyme)	Volume (with lysozyme)	Volume (with lysozyme + EDTA)
ddH2O	–	–	2.05 μL	1.95 μL	1.92 μL
10x lysis buffer (Takara)	10	1×	0.26 μL	0.26 μL	0.26 μL
10x PBS	10×	1×	0.26 μL	0.26 μL	0.26 μL
Lysozyme (50 U/μL)	50 U/μL	5 U/μL	–	0.1 μL	0.1 μL
Recombinant RNase inhibitor (Takara)	N/A	N/A	0.03 μL	0.03 μL	0.03 μL
EDTA	50 mM	0.5 mM	N/A	N/A	0.03 μL
Total volume	N/A	N/A	2.6 μL	2.6 μL	2.6 μL

• The treatment of cells with RNA*later* can hamper the MATQ-sep protocol (steps 7-18). We recommend testing different concentrations of RNA*later* with respect to both transcriptome stabilization and MATQ-seq efficiency.
• Generally, long-term storage of the PCR-amplified, but unpurified, single-cell cDNA libraries at −20°C (step 16) may result in their partial degradation. Therefore, make sure to proceed to the AMPure bead clean-up within 2 days after completion of the amplification.

### Problem 4

cDNA libraries contain mostly primer dimers, as visualized in Bioanalyzer traces as peaks below 150 bp (step 18c).

### Potential solution

• Reduce the concentrations of primers in the MATQ-seq protocol. However, we generally recommend a minimal concentration of 10 μM.
• Alternatively, the problem could be caused by inefficient bead clean-up. Therefore, ensure that the ethanol has completely evaporated before resuspending the pellet. However, be cautious that the beads will not over-dry and show no cracks.

### Problem 5

Nextera cDNA libraries have a low concentration (<2 ng/μL) (step 24c).

### Potential solution

• Measure the concentration of cDNA libraries before the Nextera protocol (step 19a) on a Qubit. Make sure that the libraries are properly diluted to 0.4 ng/μL.
• Doublecheck that the Nextera Tagment enzyme is active by performing the reaction using the positive control as the input.
• Do not tagment the library for more than 10 min. Otherwise, this could result in over-tagmentation.
• Repeat the PCR, using fresh aliquots of the Nextera PCR master mix and UDI primers.
• Since pipetting small volumes is error-prone, make sure that your pipets are accurately calibrated.

### Problem 6

The library concentration after DASH is below 2 ng/μL (step 26m).

### Potential solution

• Check the concentration of cDNA libraries before the second PCR (step 26j) on a Qubit and ensure that the libraries are properly diluted to 0.5 ng/μL.
• Repeat the PCR, ensuring that the polymerase is functional and the primers are properly diluted. Consider using fresh aliquots of PCR reagents.

## Resource availability

### Lead contact

Further information and requests for resources and reagents should be directed to and will be fulfilled by the lead contact, Alexander J. Westermann (alexander.westermann@uni-wuerzburg.de).

### Technical contact

Technical questions on executing this protocol should be directed to and will be answered by the technical contact, Elise Bornet (elise.bornet@yahoo.fr).

### Materials availability

This study did not generate new unique reagents.

### Data and code availability

“Mini-bulk” data (from small *B. thetaiotaomicron* populations consisting of 100 cells each) generated using this protocol have previously been published in Bornet et al.^1^ and are available at Gene Expression Omnibus (GEO): GSE289312. The single-bacterium RNA-seq data presented here have not previously been published and are available at GEO: GSE322907.

## Acknowledgments

We thank Antoine-Emmanuel Saliba, Jörg Vogel, Fabian Imdahl, and Christina Homberger for consultation and helpful discussions, and Lars Barquist and Laura Jenniches for help with the single-bacterium RNA-seq data analysis. We are grateful to the Single-cell Center and the core facility Systems Medicine of the Medical Faculty of the University of Würzburg for their support in library preparation and sequencing.

This project was funded by the 10.13039/501100001659Deutsche Forschungsgemeinschaft (DFG, German Research Foundation) through the Centre for Microbial Single-cell RNA-seq (MICROSEQ; 93/1105-1) and the Cluster for Nucleic Acid Sciences and Technologies – NUCLEATE (EXC 3113/1, project-ID 533767322), and by the 10.13039/501100000781European Research Council (ERC Starting Grant no.101040214).

## Author contributions

E.B. performed experiments. A.J.W. supervised the project and secured funding. Both authors wrote the manuscript together.

## Declaration of interests

The authors declare no competing interests.

## Supplemental information

Table S1. DNA oligonucleotides to in vitro-transcribe single-guide RNAs for Bacteroides thetaiotaomicron ribosomal sequences, related to Step 25 This table contains the different oligonucleotides to order as a pool from IDT. Each oligonucleotide sequence contains, in 5′-to-3′ direction, the T7 promoter (TTCTAATACGACTCACTATA), the unique gRNA sequence, the scaffold (GTTTTAGAGCTAGAAATAGCAAGTTAAAATAAGGCTAGTCCGTTATCAACTTGAAAAAGTGGCACCGAGTCGGTGCTTTTTT), and the sequence for the fill-in reaction (GGCATACTCTGCGACATCGT), as described in.^27^

Table S2. Template to calculate molarities and to pool cDNA samples, related to Step 26 This table serves as a template to calculate Cas9, sgRNA, and cDNA library molarities necessary to perform the ribosomal RNA depletion via DASH (step 26 of this protocol). Fields marked in pink should be adjusted by the user as required.
